# Friendships in Children with Williams Syndrome: Parent and Child Perspectives

**DOI:** 10.1007/s10803-022-05807-5

**Published:** 2022-11-18

**Authors:** Amanda E. Gillooly, Deborah M. Riby, Kevin Durkin, Sinéad M. Rhodes

**Affiliations:** 1 Present Address: School of Health and Wellbeing, Gartnaval Royal Hospital, University of Glasgow, 1055 Great Western Road, Glasgow, G12 0XH Scotland, UK; 2 Department of Psychology, Durham University, South Road, Durham, DH1 3LE England, UK; 3 School of Psychological Sciences and Health, University of Strathclyde, 40 George Street, Glasgow, G1 1QE Scotland, UK; 4 Child Life and Health, Centre for Clinical Brain Sciences, Royal Hospital for Children and Young People, University of Edinburgh, Edinburgh, EH16 4TS Scotland, UK

**Keywords:** Williams syndrome, Friendships, Social inclusion, Social skills

## Abstract

Although children with Williams syndrome (WS) are strongly socially motivated, many have friendship difficulties. The parents of 21 children with WS and 20 of the children themselves participated in a semi-structured interview about the children’s friendships. Parents reported that their child had difficulties sustaining friendships and low levels of interaction with peers. Barriers to friendships included difficulties with play and self-regulating behaviour. However, there was within-group variability, with a small number of children reported to have strong friendships. While parents reported friendship challenges, all of the children named at least one friend, and most said that they had never felt excluded by their peers. Future research is needed to determine optimal ways to support children with WS in their friendships.

**Supplementary Information:**

The online version contains supplementary material available at 10.1007/s10803-022-05807-5.

Williams syndrome (WS) is a genetic condition that arises from the hemideletion of approximately 28 genes from one copy of chromosome 7q.11.23 (Haas et al., 2014) and is reported to affect between 1 in 7500 and 20,000 individuals (Sparaci et al., 2012). Characteristic traits associated with the condition include mild to moderate intellectual disability (Searcy et al., 2004), an uneven cognitive profile characterised by relative strength in verbal language skills and weakness in visual-spatial processing (Rhodes et al., 2010), and differences in social interactions (Järvinen et al., 2013).

Many individuals with WS are reported to show a ‘hypersocial’ profile and a predisposition to seek out social contact (Järvinen-Pasley et al., 2010; Jawaid et al., 2012). These social interactions can be indiscriminate in nature, aligning with evidence of poor stranger danger awareness (Fisher et al., 2014; Riby et al, 2014b, 2017). This hyper-sociability has been reported to often occur alongside challenges within social cognition and social communication skills, impacting on the individual’s ability to engage in successful reciprocal social interactions (Klein-Tasman et al., 2011). These can include, but are not restricted to, difficulties sustaining reciprocal communication often associated with a reliance on stereotypic speech (Laws & Bishop, 2004), lack of awareness of social norms and rules (Klein-Tasman et al., 2011), difficulties interpreting subtle social cues (Porter et al., 2008; Tager-Flusberg & Sullivan, 2000), and difficulties self-regulating social behaviours (Lough et al., 2016b). While some of these social differences are common in other neurodevelopmental disabilities, including autism, the nature of the social experiences and social skills associated with these groups are very different and syndrome specific. This is particularly evident with regard to social motivation, with WS characterised by a ‘hypersocial’ profile, while autistic children are reported to show greater social withdrawal within interactions (Jawaid et al., 2012; Lincoln et al., 2007).

Individuals with WS are also prone to experiencing heightened anxiety which may also be associated with their social functioning (e.g. Riby et al., 2014a). It has been reported that those WS individuals who experience most anxiety also show more social challenges. Furthermore, anxiety triggers may link to social experiences, including the uncertainty of new social environments and situations, sensory sensitivities, and the negative emotions of others (Royston et al., 2021).

Klein-Tasman et al. (2011) highlighted an array of social differences associated with WS by administering the Social Responsiveness Scale (SRS) to parents and teachers of children with WS (age 4–16 years). The SRS measures social and communicative functioning across five subscales: social awareness, social cognition, social communication, social motivation, and autistic mannerisms. Over 86% of children were rated by their parents as showing atypical social behaviours, with 39% of these children scoring in the severe range of social challenges. Only 5% of children were rated by their parents within the non-impaired range of functioning on social cognition, and 20% were rated by their parents within the non-impaired range of functioning for social communication skills. Parents and teachers converged in their reports that social cognition was the most impacted domain of social functioning. These findings have been replicated in a number of studies (Gillooly et al., 2020; Riby et al., 2014a). Gillooly et al. (2020) further reported a positive association between social functioning differences (as measured using the SRS) and parent and teacher rated friendship difficulties. These studies provide accumulating evidence highlighting substantial social difficulties and differences in children with WS. These social functioning differences combined with mild to moderate intellectual impairment (Searcy et al., 2004) have implications for the children’s friendships and heightened social vulnerability (Ridley et al., 2020).

## Friendships in Williams Syndrome

Friendships have been identified as an area of substantial difficulty among many WS adults (Davies et al., 1998; Elison et al., 2010). Davies et al. (1998) conducted semi-structured interviews with the parents of adults with WS (N = 62). They revealed that 98% of adults were reported to have substantial difficulties forming and sustaining friendships and two thirds of the adults were reported to be socially isolated.

Until recently, very little has been known about the characteristics of friendships in children with WS. As part of a broader study into adaptive functioning, Greer et al. (1997) obtained ratings from the parents of children with WS (N = 15; 4–16 years) on the Vineland Adaptive Behaviour Scale (Sparrow et al., 1984). Less than one third of the children in this study were reported by their parents to have social contacts or to engage in extra-curricular activities with peers, aligning with earlier findings of social isolation (Udwin & Yule, 1991; Udwin et al., 1987). Gillooly et al. (2020) recruited the parents and teachers of 21 children with WS (7–16 years), collecting quantitative information on the children’s social skills and friendships across contexts. The children with WS were reported to have significantly greater friendship difficulties than a neurotypical child population sample. Many children with WS were reported to have difficulties sustaining friendships, experience social exclusion from their peer group, and show social functioning differences including difficulties playing with peers. These social difficulties were reported by both parents and teachers suggesting that the difficulties are evident across home and school contexts.

Friendship difficulties have also been reported to be implicated in children with intellectual disabilities that are not linked with WS. Adolescents with intellectual disabilities were reported to score lower on dimensions of friendship quality, warmth and closeness and reciprocity, and to spend less time with friends compared to their neurotypical peers (Tipton et al., 2013), with these findings supporting earlier work (Solish et al., 2010; Wiener & Schneider, 2002). The researchers also found that social skills were a significant predictor of friendship difficulties in the adolescents with intellectual disabilities (Tipton et al., 2013). This suggests that intellectual disabilities and associated social skill difficulties, including difficulties identifying and responding appropriately to social cues within an interaction, may be key factors underlying friendship challenges among children with WS.

To date, few studies have gone beyond reporting prevalence data to attempt to understand the factors underlying friendship difficulties in this population. Qualitative evidence can provide richer content of the lived experience and voices of people with learning disabilities (Seale et al., 2015) and can be informative in identifying strengths and difficulties and targeting support for the friendships of children with WS. Previous research has used interviews to examine friendships in children with a range of neurodevelopmental conditions (Bagwell et al., 2001; Cuckle & Wilson, 2002; Orsmond et al., 2004; Rowley et al., 2012) but as yet, no qualitative studies have been conducted to examine friendships specifically in children with WS. Research looking at the perspectives of parents and children regarding difficulties in friendships is particularly needed and provides unique insights that will be valuable in informing future support. The current study aims to address this by examining the perspectives of children with WS and their parents using semi-structured interviews to obtain rich qualitative insights into the characteristics of friendships in children with WS.

## Method

### Participants

Twenty-one children with WS (7–16 years; M: 11.83 years; 12F, 9M) and their parents were recruited from across the UK. The families were members of the Williams Syndrome Foundation UK charity and had previously provided permission to be contacted regarding participation in research studies. All families who had a child with WS aged between 6 and 16 years, and lived in Scotland, North England, and North Wales, were contacted by letter and invited to participate in this study. 27% of the families contacted opted to participate in this study. All children had previously received a formal diagnosis of WS involving a positive genetic fluorescent in situ hybridisation test. There were no other exclusion criteria for this study.

Measures of verbal and visual-spatial performance were collected during the research visit. Verbal ability was measured using the British Picture Vocabulary Scale (Dunn et al., 1997), with a mean score of 67.50 (SD: 19.52, IQR: 51–87.5). Visual spatial performance was measured using Ravens Coloured Progressive Matrix (Ravens et al., 1998), with a mean score of 16.67 (SD: 3.79; maximum possible 36; IQR: 13.5–18.5). As expected for a WS sample, there is a clear difference between chronological age and both verbal and non-verbal ability. For receptive vocabulary using the BPVS, the mean verbal mental age for the sample was 6 years 4 months (much lower than the 11.8 year average chronological age). For non-verbal ability using the Ravens, if the sample was to perform at the equivalent level of their chronological age this would give an interquartile range of 30–34 out of 36. However, the interquartile range for the current sample was only 13.5–18.5 out of 36. Both verbal and non-verbal performance were therefore well below chronological age expectations, as expected for a WS sample. 38% of the children with WS were reported to show moderate levels of impairment in everyday reciprocal social interactions, and 48% were reported to show severe levels of impairment, as measured in the SRS 2 (Constantino & Gruber, 2005).

Fourteen of the children attended a mainstream school and seven attended a specialist provision school. Mainstream schools are general educational settings which teach pupils of a wide range of abilities. Specialist provision schools are schools which are designed to cater specifically for the needs of children with a special educational need or disability. The children in mainstream school received varying levels of additional support. Most of the children attended lessons with their neurotypical peers, receiving some additional teaching support within the classroom. A smaller number of children attended a specialist provision unit within a mainstream school. Twenty of the children took part in an interview with a researcher, with one child unable to take part due to their communication difficulties. Interviews were also carried out with the child’s mother (N = 11), father (N = 2) or both parents together (N = 8). All families were contacted through the Williams Syndrome Foundation UK.

### Measures and Procedure

Bespoke semi-structured interviews were developed by the authors. These were adapted from the friendship interview schedule used by Cuckle and Wilson (2002) in their assessment of friendships in adolescents with Down syndrome. The interview schedules were modified to tap into specific aspects of the WS social phenotype, including hypersociability and experiences of social anxiety. The parent interview schedule contained 14 items designed to capture their perspectives on six aspects of their child’s friendships: the quality of friendships, appropriateness of social interactions, level of contact with peers, social inclusion, and participation in extra-curricular clubs and activities (please see appendix 1). A 13-item interview schedule was also designed for use with the children with WS to measure their perceptions of the quality of their friendships, their extra-curricular engagement, and their understanding of the term ‘friendship’ (please see appendix 2).

Children with WS and their parents were visited at home where they participated in a semi-structured interview. Some parents were present during their child’s interview, while others were in a nearby room within the home, depending on their child’s preference. Parents did not contribute during the children’s interview. All interviews were audio recorded using a Dictaphone recording device with the permission of the participant and later transcribed verbatim. The average length of the interviews were 28 min 58 s (parents) and 7 min 1 s (children). Full ethical approval was granted from the ethics committee at the first author’s institution.

### Data Analysis

Parent interview data were analysed using the qualitative method of thematic analysis (Braun & Clarke, 2006). An inductive approach was taken, where the themes were driven by the data. Thematic analysis was conducted within a realist paradigm, where the data were interpreted as a direct communication of the participants’ thoughts, attitudes and motivations (Braun & Clarke, 2006). In line with the guidance of Braun and Clarke, (2006), each stage of thematic analysis was followed in sequence. The audio data were initially transcribed verbatim. Initial codes were generated across the database by two of the authors (AG and SR) and points of interest noted. The authors met on several occasions to review the themes and discuss discrepancies. Through careful review and consideration, these initial codes were condensed into a final set of three themes. It was not possible to conduct thematic analysis on the children’s interview data due to the shorter nature of the responses. Key findings from the interviews with the children with WS are reported, and quotes provided where available, in line with the methods used by Ozsivadjian et al. (2012).

## Results

Three themes were identified from the semi-structured interviews with the parents of the children with WS: (1) quality of friendships concerns the quality of time spent with friends and the children’s ability to sustain friendships across time, (2) barriers to friendship development includes the children’s difficulties self-regulating behaviour, difficulties with play, and the role of social anxiety, and (3) impact of the peer group on the friendship dynamic concerns differences in the nature of the children’s friendships with neurotypical peers, and the role of shared interests and abilities.

### Quality of Friendships

Eight (out of 21) parents reported that their child with WS had difficulties sustaining friendships with their peers: “he flits from people to people. He doesn’t have anybody that he repeatedly talks about or anything like that” (p4, Male, age 13). Three of these parents noted that their child believed that they had a best friend but this friendship was not reciprocated: “she’s got lots of friends who she thinks are all her best friends. That can be from people she’s met at primary school to someone she’s met 5 min ago” (p2, Female, aged 14).

Ten (out of 21) parents reported that their child never or only on a few occasions had play dates with peers outside of school: “he doesn’t socialise out of school, so he never has anybody back for tea, he is never invited to other people’s for tea, it just doesn’t happen” (p4, Male, age 13). Another parent reported that “It’s most noticeable when invites have been handed out in the class and it can be very difficult to explain to her then that no you haven’t got an invite. I think that she can find upsetting and the fact she’s not been included in things that she perhaps wants to be. Other times I think it’s less obvious as she doesn’t necessarily see the other children going home and having playdates after school” (p11, Female, age 8). Three parents made comparisons between their child’s friendships and the friendships of their typically developing siblings to further highlight their difficulties: “When you’ve got another child you know the difference, my other daughter comes home and she asks almost every night can such a person come on Thursday and can I go here and do that and that. It’s different isn’t it?” (p8, Female, age 13).There was, however, diversity in individuals’ experiences of friendships and five parents reported that their child had strong reciprocal friendships and regular contact with peers: “She has a couple of really good friends. She’s very popular, especially around here” (p6, Female, age 7).

### Barriers to Friendship Development

Parents also discussed their children’s social functioning differences as a barrier to their friendship development. Nine (out of 21) parents reported that their child showed difficulties self-regulating their behaviour, including disinhibited tactile behaviour and a poor awareness of personal space boundaries: “Yeah because he would just wrap his arms around their neck and not let them go. You would have to go and pull him off and say ‘leave them alone’” (p10, Male, age 12). One parent reported “she gets a bit obsessional as in she’ll maybe be on her messenger thing and I’m always checking it and have noticed that she has sent them a message like 15 times in a row, like every minute, ‘Hi, Hi, Hi, Hi’ waiting for them to reply” (p2, Female, age 14). Another parent indicated her struggle to moderate her child’s behaviour through repeated reminder and education regarding appropriate behaviour; however, noted that despite these efforts he was often unable to control these actions: “He gets fixations on certain people sometimes…now we have a conversation every day that you can say hello to her but you don’t follow her around…but unless you say that to him all the time he will forget and do it again” (p12, Male, age 9). Six parents raised concerns that their child’s disinhibited behaviour was sometimes overwhelming or uncomfortable for their peers: “they don’t know how to react to it and deal with it and kind of get scared off a little bit” (p4, Male, age 13).

Parents also discussed their child’s difficulties engaging in play with peers. Seven (out of 21) parents noted that their child did not play interactively with their peers, either choosing to engage in solitary interests, or observing other children play: “he didn’t play with any of them, he was just playing alone and is happy to do so… didn’t play with a single person there or talk to them or anything really but he had a great party” (p20, Male, age 11). Another parent reported that her son “would be more on the periphery. Things like if they are playing a computer game, he is happier just to watch than to take part” (p3, Male, age 11). Eight (out of 21) parents reported that their child often lost interest during play with peers. Parents described occasions where peers from school came to visit and their child had lost interest in playing with their peers and retreated back to his or her own solitary activities: “she could still abandon a friend really, have someone round, be really delightful that they are there and then abandon them and gravitate back to her solitary interests” (p21, Female, age 15).

Six (out of 21) parents also discussed their child’s experience of social anxiety: “She gets anxious, we had quite a lot of people here for her birthday and she was fine, it was great, and then when she’s ready for everybody to go, she just bursts into tears and goes upstairs. So it must be an anxiety that builds” (p5, Female, age 15). Another parent reported: “He ends up crying and it’s overwhelming. Yeah he can’t manage, can’t cope. Like when we went to Christmas parties before Christmas, I ended up leaving with him because he was crying” (p15, Male, age 9).

### Impact of the Peer Group on the Friendship Dynamic

Another prominent theme across the interviews was the impact of the child’s peer group on the dynamic of their friendships. Five (out of 21) parents noted that typically developing peers often took on an older sibling or caring role within the friendship: “they get on brilliant and are very close but it’s not a friendship where, they wouldn’t go out and do a girlie shopping day or anything like that. She looks after her…more of a carer than a friend” (p8, Female, age 13).

While friendship difficulties were reported across the sample, irrespective of age, ten (out of 21) parents noted that their children’s friendships with their neurotypical peers had become more challenging with age as their peers gained increased levels of independence from their parents and as differences in interests became more apparent: “They are away far ahead now, I mean they are probably out socialising in places that they shouldn’t be but that’s what kids do but there’s no way that she could be put in that situation” (p2, Female, age 14). Another parent highlighted the difference in her daughter’s support needs compared to her neurotypical peers, with this becoming more apparent with increasing age: “I think she is just out of sync and they have all become quite independent and so getting public transport. I think when it got to the stage where the parents were no longer organising their social activities …. they are able to go to the swimming pool on their own or go to the park on their own but she just couldn’t do that without adult support and so I think as that began to happen it was a struggle to maintain” (p21, Female, age 15). Some parents also discussed a difference in interests between their child and their neurotypical peers:“the children are getting more grown up and they like playing on X-BOX and computer games and they’ve got these shared interests, whereas he doesn’t do that, he doesn’t like, you know he’s not got the coordination or he wouldn’t know how to play one of those games, so really he’s got nothing in common with children his age” (p12, Male, age 9).

Three parents noted that their child’s shared interests and similar ability levels with their peers with disabilities had led to the development of friendships: “I dropped him off at youth club the first couple of times and he is just fully part of it… Yeah, he’s found his little niche in the world” (p4, Male, age 13). However, there were individual differences in the children’s experiences, with some children experiencing challenges when socialising with peers with disabilities: “They don’t mix together very well, although they want to be friends but they don’t know how to be friends as both of them have got social and emotional immaturity…they don’t know how to play without being led a bit, and if there’s no- one else in the group with the maturity to lead them, they just clash” (p7, Male, age 8).

### Interviews with Children with WS:

All of the children named at least one close friend and listed activities which they took part in with their friends. This included sports, technology based games, and general play. However, some children (N = 6) said that they never saw their friends out of school. A few children (N = 4) noted that their friends looked after them. One child said that friends were important to her because “they look after me” (p11, Female, age 8), with another child reporting that the other children in his class “always care for me” (p7, male, age 8). Most of the children (N = 16) said that they had never been left out by their peers. Among the children who reported to being left out by peers, most reported that this only happened occasionally or referred to one specific child. However, one boy reported “they don’t come over to me” and that they left him out “because they aren’t kind” (p12, male, age 9). Seven children described friendships as engaging in activities together, while ten children referred to positive character traits that make someone a friend e.g. “when they are kind and they care about you. They help you when you’re down” (p5, Female, age 15). Another child reported “If you are looking out for them and looking after them and see if they are OK, and if not you can speak to them and see what’s been going on and sort it out” (p14, female, age 16). 3 children were unable to define friendship.

## Discussion

The current findings provide evidence of substantial friendship challenges for children with WS as derived from parents’ qualitative insights. A clear focus of the parental reports was that their child struggled to sustain friendships and many had limited interaction with their peers. While the children with WS all named at least one friend, these peer interactions were often reported to largely take place in school. Parents discussed barriers to their child’s friendships including difficulties regulating behaviour, differences in play, and the child’s experiences of social anxiety. The children’s peer group was also reported to have an impact on the dynamic of their friendship. The gap between the child with WS and their neurotypical peers was reported to expand with age as their peers gained increased independence, making it substantially more challenging to sustain these friendships. However, it is important to note that there was variability among the children, in line with the heterogenous nature of WS, with a small number of children having strong friendships.

Aligning with previous research (Gillooly et al., 2020; Greer et al., 1997), difficulties sustaining friendships and low levels of interaction with peers were raised by parents as areas of significant difficulty. These difficulties were reported for both children and adolescents, suggesting that this may be a difficulty that transcends development. While parents perceived that their child had challenges with friendships, all of the children with WS named at least one friend, and most children said that they had never felt excluded by their peers. The different perceptions of the children with WS and their parents is interesting, suggesting that there are differences in what friendships mean to children with WS, and how they experience friendship. This highlights the value of a multi-informant research design, with insight into the lived experiences of the children being critical in informing their future support needs.

Some parents noted that while their child believed that they had a best friend, parents perceived that this friendship was not always reciprocated by their peer. This finding suggests that children with WS have difficulties understanding the complexities of friendships at this age, mapping onto a profile of social-cognition difficulties in WS (Plesa-Skwerer et al., 2006). Several barriers in sustaining friendships were raised, including the children’s disinhibited tactile behaviours and difficulties engaging in play with their peers. These difficulties can be mapped back to a profile of executive function and sensory processing differences in WS (Glod et al., 2020; Rhodes et al., 2010). Children with WS showed difficulties regulating their behaviour during social interactions with their peers and were reported to engage in inappropriate tactile behaviour and poor personal space regulation (cf. Lough et al., 2016a). This is likely to be accounted for by response inhibition difficulties in WS, in line with the theoretical assumptions of the frontal lobe hypothesis (Little et al., 2013). Parents believed that some peers were uncomfortable and overwhelmed by their child’s disinhibited nature, and this negatively impacted their peer relationships. This links back to social-cognition difficulties, where it was clear the children were not able to identify their peers’ discomfort. The current findings build on previous evidence of disinhibited tactile behaviour with peers (Gillooly et al., 2020) by using qualitative insights to understand the implications of this behaviour for the children’s friendships.

Children with WS had difficulties engaging in play with their peers, aligning with previous evidence (Fanning et al., 2021; Gillooly et al., 2020). While neurotypical children transition through a phase of solitary play during the pre-school years (Parten, 1932), WS children and adolescents of different ages were shown to engage in high levels of solitary play. The children with WS often had different interests from their peers that would not be considered typical for their age, aligning with existing evidence of restrictive interests and repetitive behaviours in WS (Royston et al., 2018) that is commonly reported in autistic children (Halsall et al., 2021). Executive function differences (Leyfer et al., 2006), reduced intellectual capacity (Searcy et al., 2004), and restrictive and repetitive interests (Royston et al., 2018), are all likely to contribute to challenges in play for children with WS. With evidence supporting the role of shared interests in friendship development (Gifford-Smith & Brownell, 2003), this can be suggested to be a contributing factor to the children’s friendship difficulties.

Some children with WS benefited from socialising with peers who shared similar interests and levels of social and intellectual functioning. The parents of these children believed that this promoted more opportunities for their child to build friendships. However, there were individual differences and some parents reported that their child’s friendship difficulties persisted, or in some cases were heightened when socialising with peers with disabilities. These parents highlight the value of a level of compatibility within a friendship, with several of the children with WS facing challenges due to their own unique requirements. These findings again highlight the importance of an individual person centred approach given the heterogeneity observed within this population.

Experiences of social anxiety were reported in the present study, with the children with WS reported to becoming emotionally overwhelmed in certain social situations, posing challenges for their friendships. The children’s experiences of social anxiety may contribute to their difficulties within key social functioning domains, as they may be less able to pick up on important social cues within interactions (Riby et al., 2014a), increasing their social vulnerability and impacting on their ability to build and sustain friendships.

A common theme across the interviews was the children’s social vulnerability. Among the children who attended mainstream school, although neurotypical peers were generally very caring towards the child with WS, friendships had become more difficult to sustain with increasing age arising from their peers’ increased social independence, progressive maturity and the development of new interests with age. The impact of the children’s intellectual disabilities on their friendships is suggested to become more apparent with age as the gap between the child with WS and their neurotypical peers widens. With the reducing need for parental supervision among neurotypical peers, the need to exercise appropriate social judgment becomes particularly crucial as children are exposed to a wider range of social situations. The children’s difficulties self-regulating behaviour during social interactions, combined with evidence of poor stranger danger awareness (Riby et al., 2014b) and reduced intellectual capacity (Searcy et al., 2004), are likely to make sustaining friendships increasingly challenging and may increase vulnerability to peer victimisation (Ridley et al., 2020).

There were individual differences in the children’s experiences of friendship. A small number of parents reported that their child had a strong reciprocal friendship with one or more peers. These parents noted that their child was able to sustain friendships and was well integrated in their peer group. These children were also reported by their parents to be able to sustain reciprocal conversations with their peers and to exercise an awareness of social norms and boundaries within their peer interactions. This suggests, in line with previous evidence by Gillooly et al. (2020), that these social functioning skills play a key role in the friendships of children with WS. These reports build on previous findings on the heterogeneous nature of cognition and behaviour in WS (Little et al., 2013; Porter & Coltheart., 2005). It is important that this within-syndrome variability is explored to understand the factors underlying friendship difficulties in WS.

### Limitations and Future Research

It is important to acknowledge limitations of the current study. The sample size in the current study (N = 21) is in line with previous WS studies (Martens et al., 2008) and sample sizes used in qualitative research (Fisher et al., 2017; Lough et al., 2016b). However, as with all qualitative research, caution should be taken when generalising the current findings to the wider WS population, considering within-syndrome variance in these experiences. While we collected initial data on the children’s social skills and their verbal and non-verbal performance, information was not collected on co-occurring diagnoses or levels of anxiety. It is possible that these may have an effect on the children’s friendships and social skills and this should be explored within future studies. The current paper reports on the perceptions of children with WS and their parents on the children’s friendships. This study provides interesting preliminary insights into the children’s understanding of their own friendships. It was not possible to conduct an in depth qualitative analysis on this data due to the shorter nature of the responses. Future research should explore this further by using a range of accessible participatory methods to further capture the voices of the children themselves and better understand what is important to children with WS within a friendship. Future research is also needed to identify optimal ways to support children with WS in their social interactions, targeting the identified areas of difficulty, while considering the evident individual variability. 

## Conclusion

This is the first in depth qualitative study to examine the factors underlying friendship difficulties in children with WS, obtaining the perspectives of both children with WS and their parents. The current study provides evidence of substantial friendship challenges for children with WS. Parents reported on their child’s difficulties sustaining friendships, their low levels of interaction with peers and exclusion from peer activities. In line with the heterogeneous nature of WS, there was within-syndrome variance in these experiences with a few children reported to have strong friendships. While parents perceived that their child had challenges with friendships, all of the children with WS named at least one friend, and most children said that they had never felt excluded by their peers.

Disinhibited tactile behaviours, differences in play, and social anxiety were identified as barriers to friendship development. These findings map onto the broader WS phenotype, specifically the children’s social vulnerabilities and unique social cognition, sensory, and executive function profiles. These findings build substantially on previous work, providing rich qualitative insights into the children’s friendship experiences and challenges. The current findings in conjunction with previous research should be used to inform the design of interventions to target social functioning.

### Supplementary Information

Below is the link to the electronic supplementary material.

Supplementary file1 (DOCX 18 kb)
