# Supplementary material for: Friendships in Children with Williams Syndrome: Parent and Child Perspectives
Source: J Autism Dev Disord. 2022 Nov 18;54(2):509–17. doi: 10.1007/s10803-022-05807-5 (PMC10821955; doi:10.1007/s10803-022-05807-5)
Supplement: Supplementary file 1 — Supplementary file1 (DOCX 18 kb) [file 10803_2022_5807_MOESM1_ESM.docx]

Appendix 1: Friendship interview schedule (parents)

### In your opinion, do you consider your child to have a best friend?

[If yes: How does your child know this person? Does this person have a disability? In your opinion is this friendship reciprocated by this person?

If no: Does your child have any friends or acquaintances that he/she plays with and/or talks to?]

### Can you tell me anything about your child’s ability to make friends? [Are there any examples which you feel highlight this?]

### When it comes to activities with peers, do you ever find the situation where your child is left out to a certain extent? [If you don’t mind elaborating, are there any examples which spring to mind? Do you feel that your child notices this/feels left out?]

### In your opinion, do you think that your child is drawn to certain age groups when initiating contact with others? [Does he/she initiate contact with both children and adults equally? Are there any examples which illustrate this?]

### Are there any situations where your child has been picked on by peers?

1. Does your child experience anxiety in social situations? [Are there any examples of this which spring to mind? In your opinion, has this anxiety affected his/her social relationships?]
2. Does your child demonstrate any of the following behaviours when in social situations? Excessive chatter, intense behaviour towards others, asking inappropriate questions? [Are there any examples which spring to mind? Do you feel that any of these behaviours affect his/her social relationships?]

### Can you tell me about your child’s ability to hold conversations with other people?

1. Does your child understand that his/her friends may also have others friends who are not in the same group whom they spend time with? [Does this upset your child? Is he/she able to play by himself/herself if these friends are unavailable?]

### Does your child engage in any of the following activities with peers? (Computer/video games, listening to or making music, engaging in sports, general play or anything else?).

### [If yes, how often does your child engage in these activities?

### If no, how does your child spend his/her free time?]

### Does your child attend any organised clubs/activities? [Does he/she enjoy these? Is he/she included by the other children e.g. in teams, playing, talking? Does he/she experience any difficulties at these clubs?]

12a. How often, if ever, does your child get invited to the homes of other children from school or in the neighbourhood?

12b. How often, if ever, does your child get invited out to activities by other children’s parents e.g. dinner, the cinema, bowling?

12c. How often, if ever, does your child get invited to the birthday parties of other children?

13. Does your child often go up to other children and hug them or hold their hand? [Has this had any positive or negative effects to his/her social relationships?]

14. Do you have any further comments on the quality of your child’s social life and your hopes/concerns for his/her future?

Appendix 2: Friendship interview schedule (child with WS)

### Do you have a best friend? [If yes: How do you know this friend?]

### Are the other children in school nice?

- 1. What do you like to do with your friends?

[Do your friends come over to your house?

Do you go over to their house?

Do you play inside or out in the garden/street?

Do you sometimes go out with your friends and their families? What do you do?

What’s your favourite game to play with friends?]

### Do you go to any clubs?

### [Do you like these clubs?

### What is your favourite thing about these clubs?

### Do you have friends that you talk to at the clubs?

### Do you have friends that you play with at the clubs?]

### One girl told me that she argues with her friends sometimes. Do you ever fall out or argue with your friends? [When does this happen? How does this make you feel?]

### One boy told me that his friends said that he couldn’t play football with them. Do your friends ever tell you that you can’t play with them? [Does this happen a lot or just sometimes? How does this make you feel?]

### Do you like talking to the teachers at school?

- 1. Do you like talking to the children in your class? [What kind of things do you talk about?]
  2. What do you do at break time at school? [Do you spend break time with the other children? Who do you talk to?]
  3. What do you do at lunch time at school? [Do you spend lunch time with the other children? Who do you eat lunch with? Who do you talk to?]

### What makes a good friend?

### What do you like about your friends?

### Do you think friends are important? [If yes, why are they important?]
